# Supplementary material for: Antipsychotics Affect Satellite III (1q12) Copy Number Variations in the Cultured Human Skin Fibroblasts
Source: Int J Mol Sci. 2023 Jul 10;24(14):11283. doi: 10.3390/ijms241411283 (PMC10380077; doi:10.3390/ijms241411283)
Supplement: Supplementary file 1 [file ijms-24-11283-s001.zip › Supplement S1-S4.pdf]

Supplement

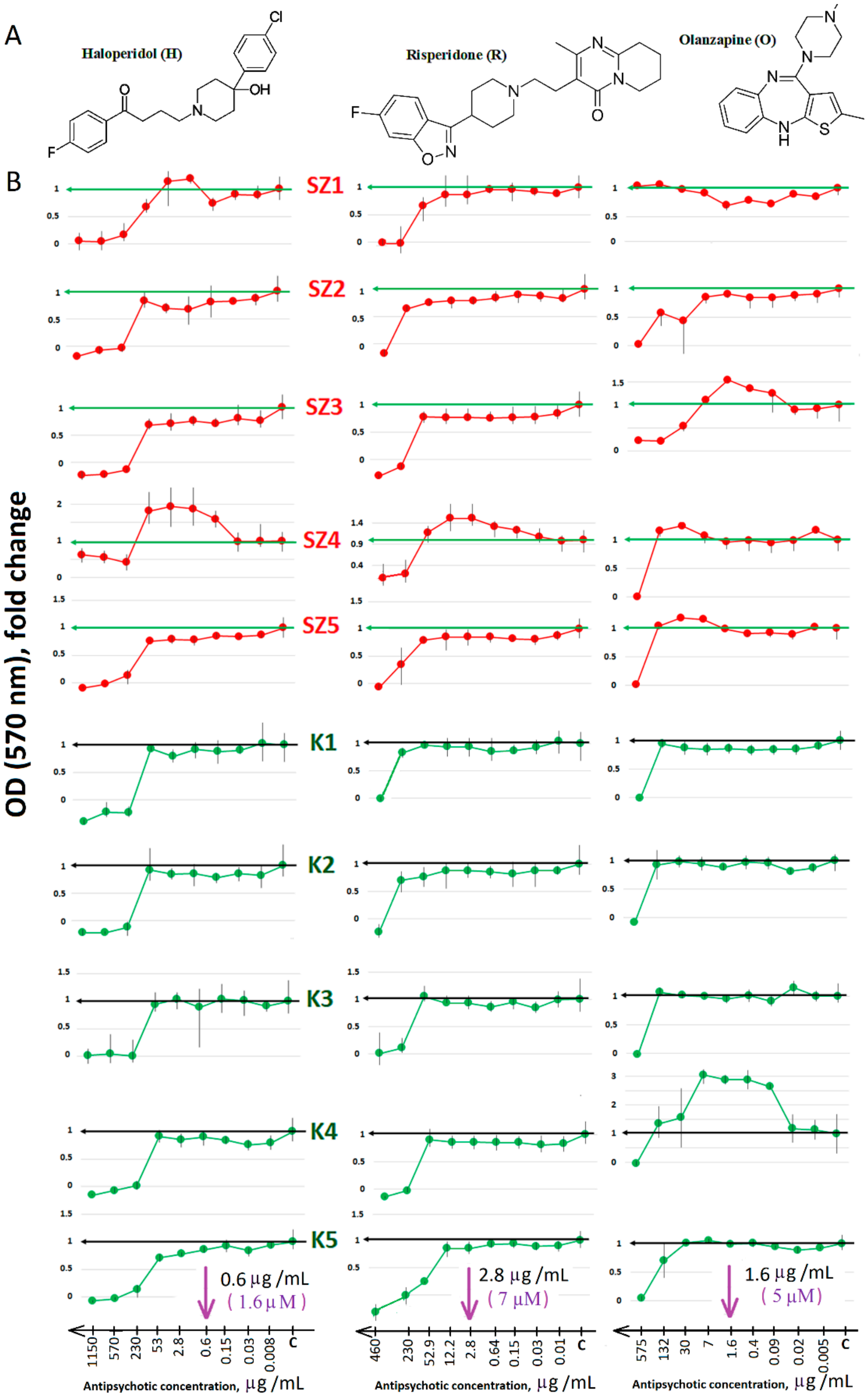

Figure S1. Determination of the non-toxic antipsychotic concentrations A. The structural formulas of the antipsychotics. B. The results of the MTT test. Eight parallel wells of the plate were used for each antipsychotic concentration (SD is given). The survival analysis was conducted using 3-(4, 5-dimethylthiazol-2-yl)-2, 5-diphenyltetrazolium bromide (MTT) as described earlier [36]. The plates were read at 570 nm using EnSpire reader.

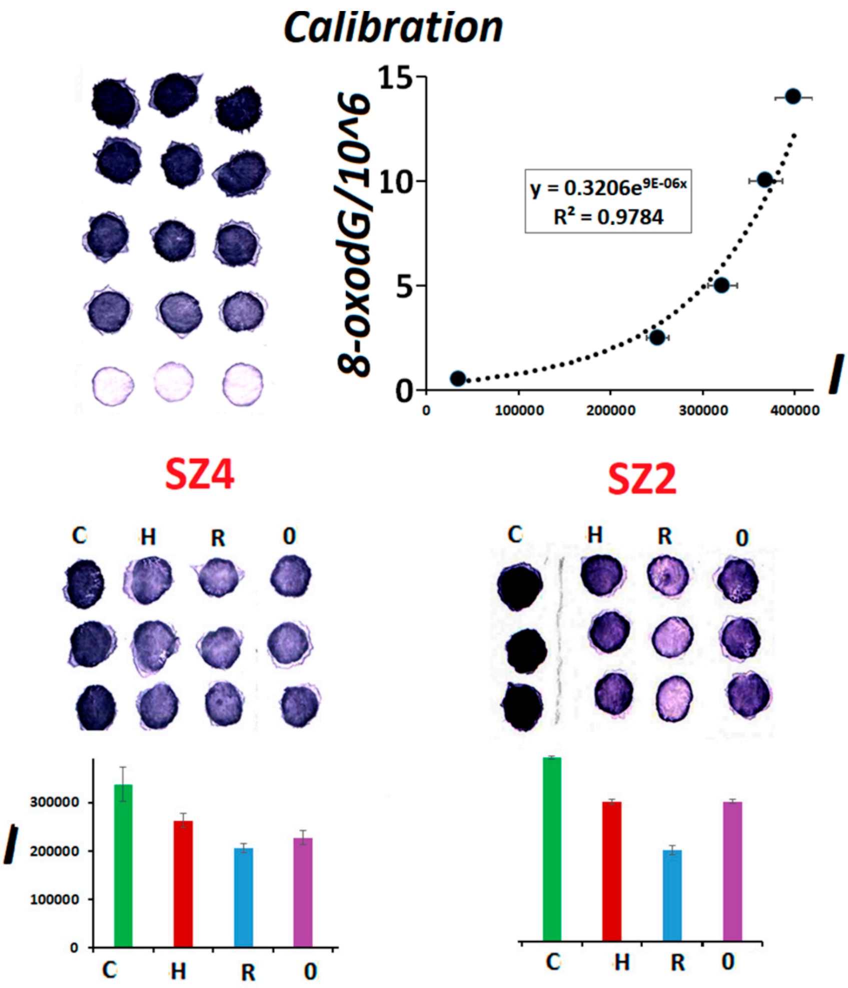

Figure S2. Measuring the DNA oxidation degree in the DNAs isolated from HSFs. Example for 8-oxodG detection in DNA.

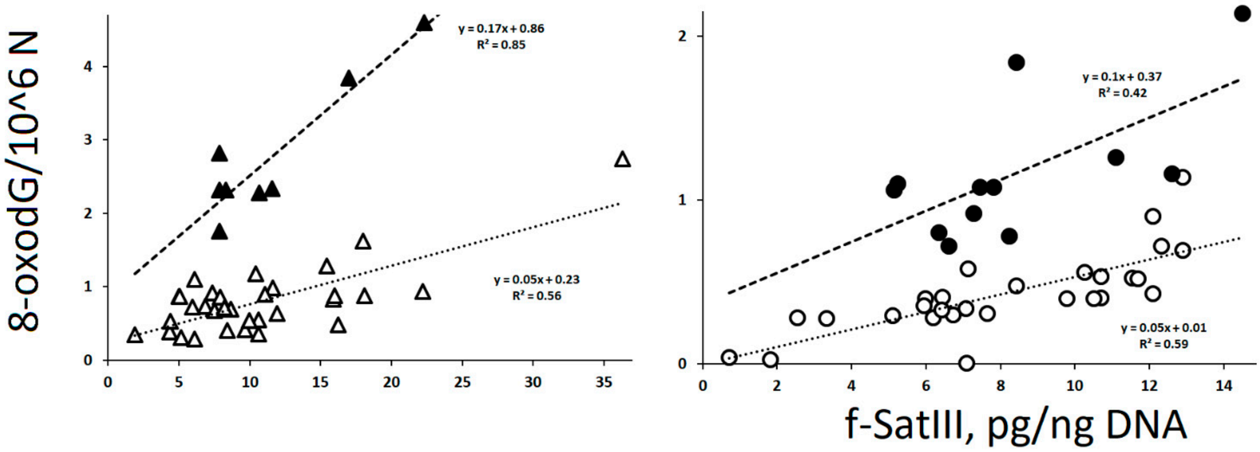

Figure S3. Dependence of the 8-oxodG content in DNA isolated from HSFs (24 h and 96 h) on the f-SatIII abundance.

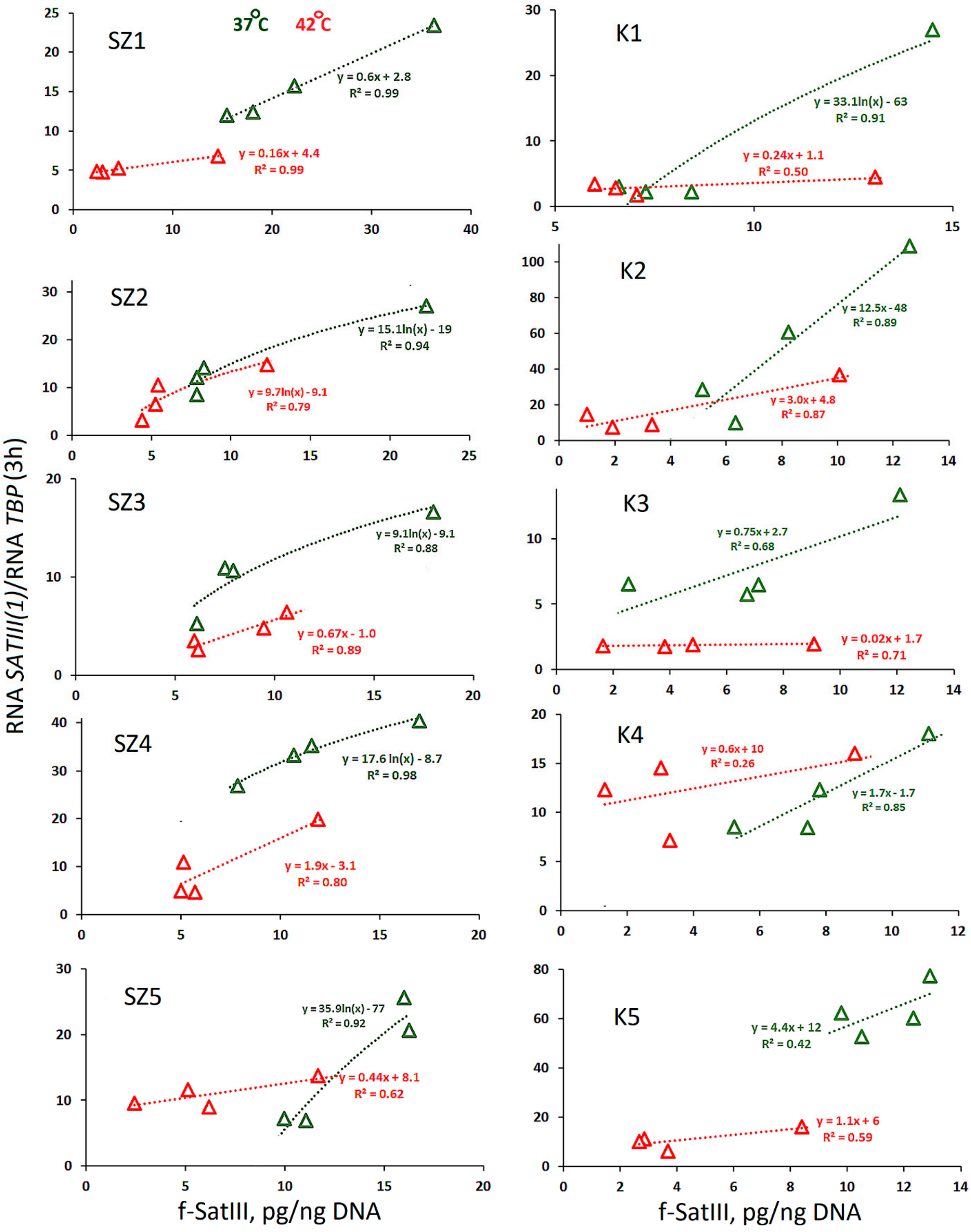

Figure S4. The effect of antipsychotics on RNA SATIII level. Green line - Dependence of the RNA SATIII level in the RNA isolated from HSFs (3 h) on the f-SatIII content in DNA isolated from HSFs (96 h). Red line - Dependence of the RNA SATIII level in RNA isolated from HSFs (42°C) on the f-SatIII content in DNA from the HSFs (42°).
