# Supplementary figures and images for: Antipsychotics Affect Satellite III (1q12) Copy Number Variations in the Cultured Human Skin Fibroblasts
Source: Int J Mol Sci. 2023 Jul 10;24(14):11283. doi: 10.3390/ijms241411283 (PMC10380077; doi:10.3390/ijms241411283)

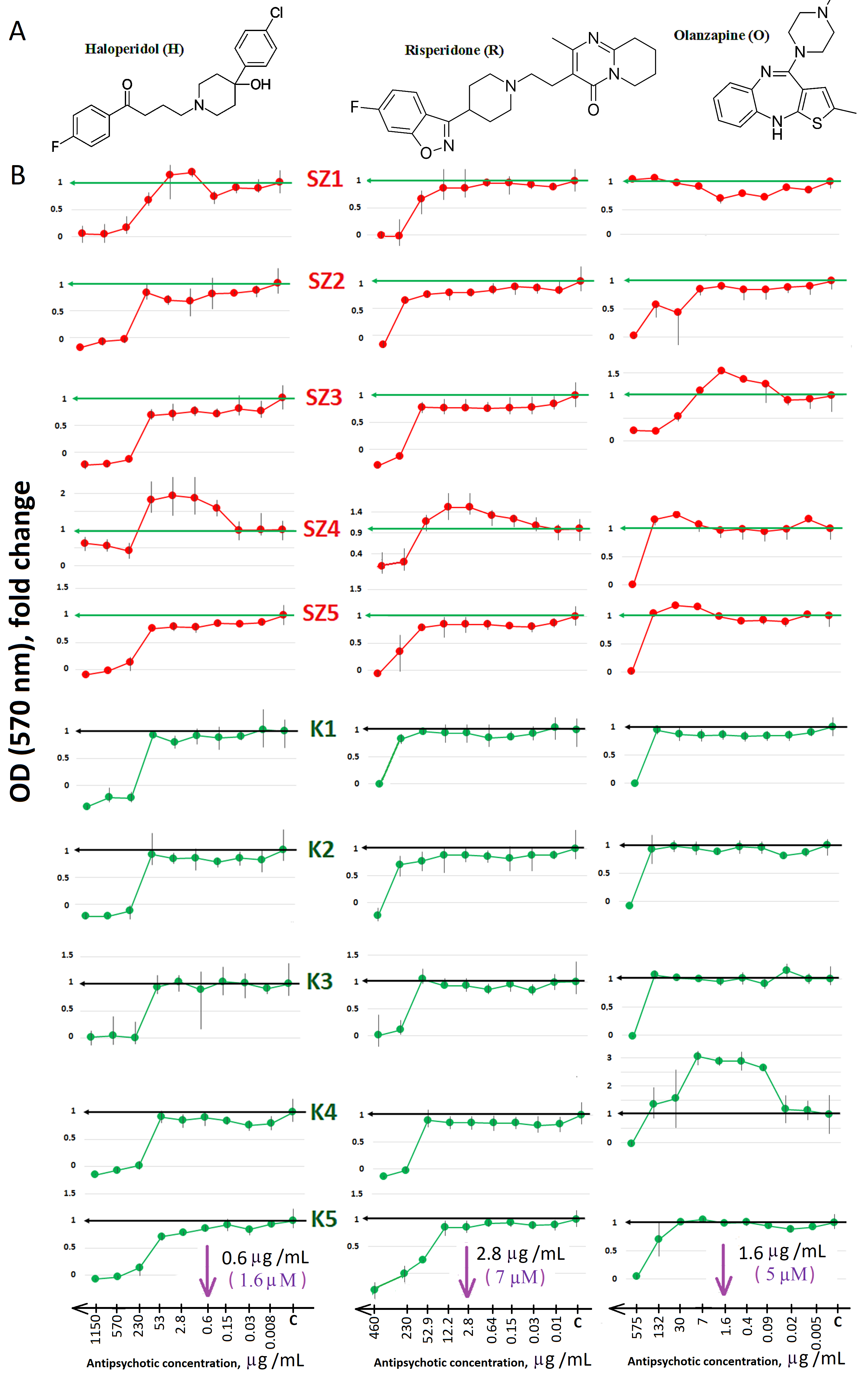

Supplement: Supplementary file 1 [file ijms-24-11283-s001.zip › Figure-1 Suppl.tif]

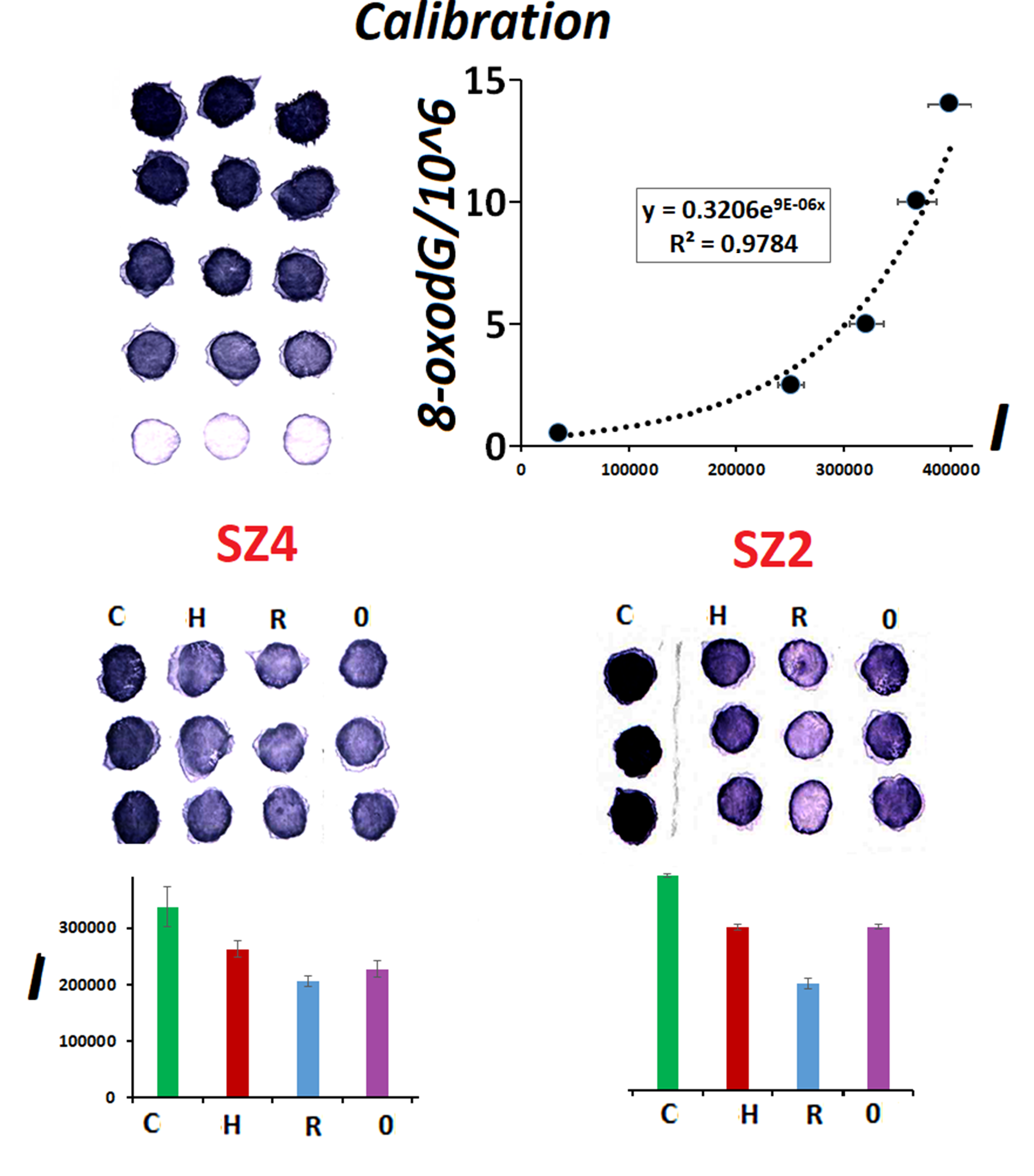

Supplement: Supplementary file 1 [file ijms-24-11283-s001.zip › Figure-2 Suppl.tif]

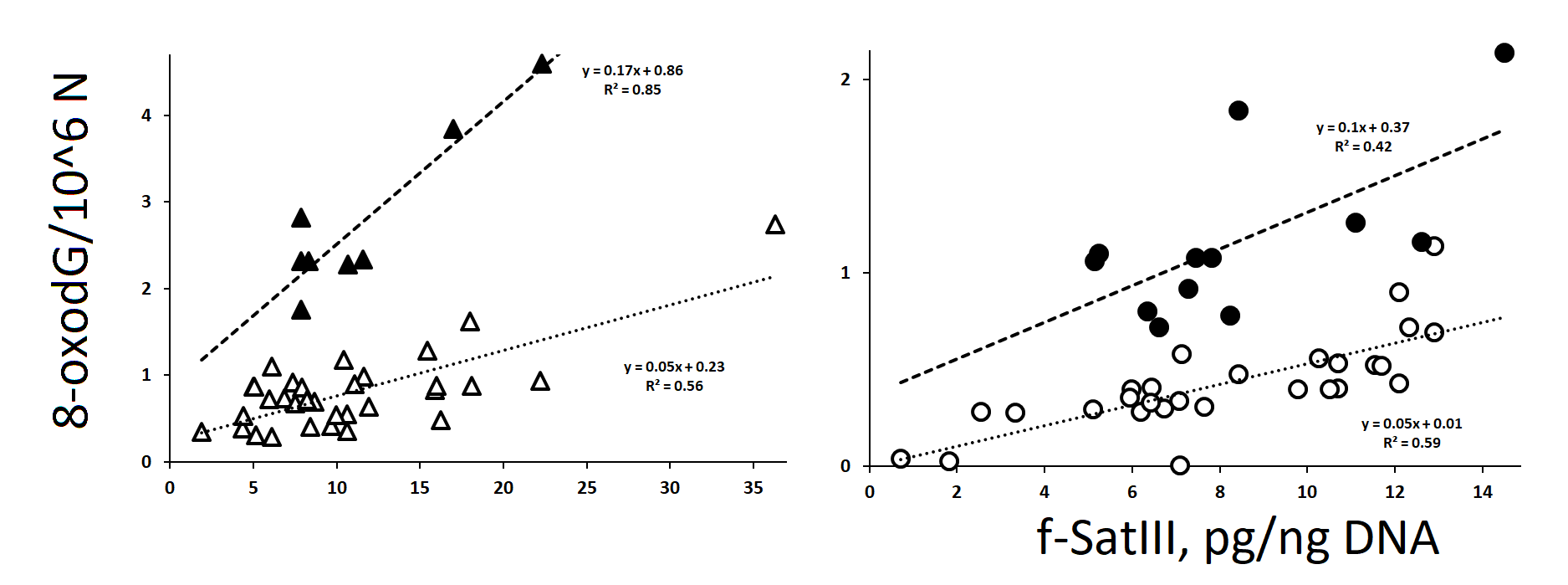

Supplement: Supplementary file 1 [file ijms-24-11283-s001.zip › Figure-3 Suppl.tif]

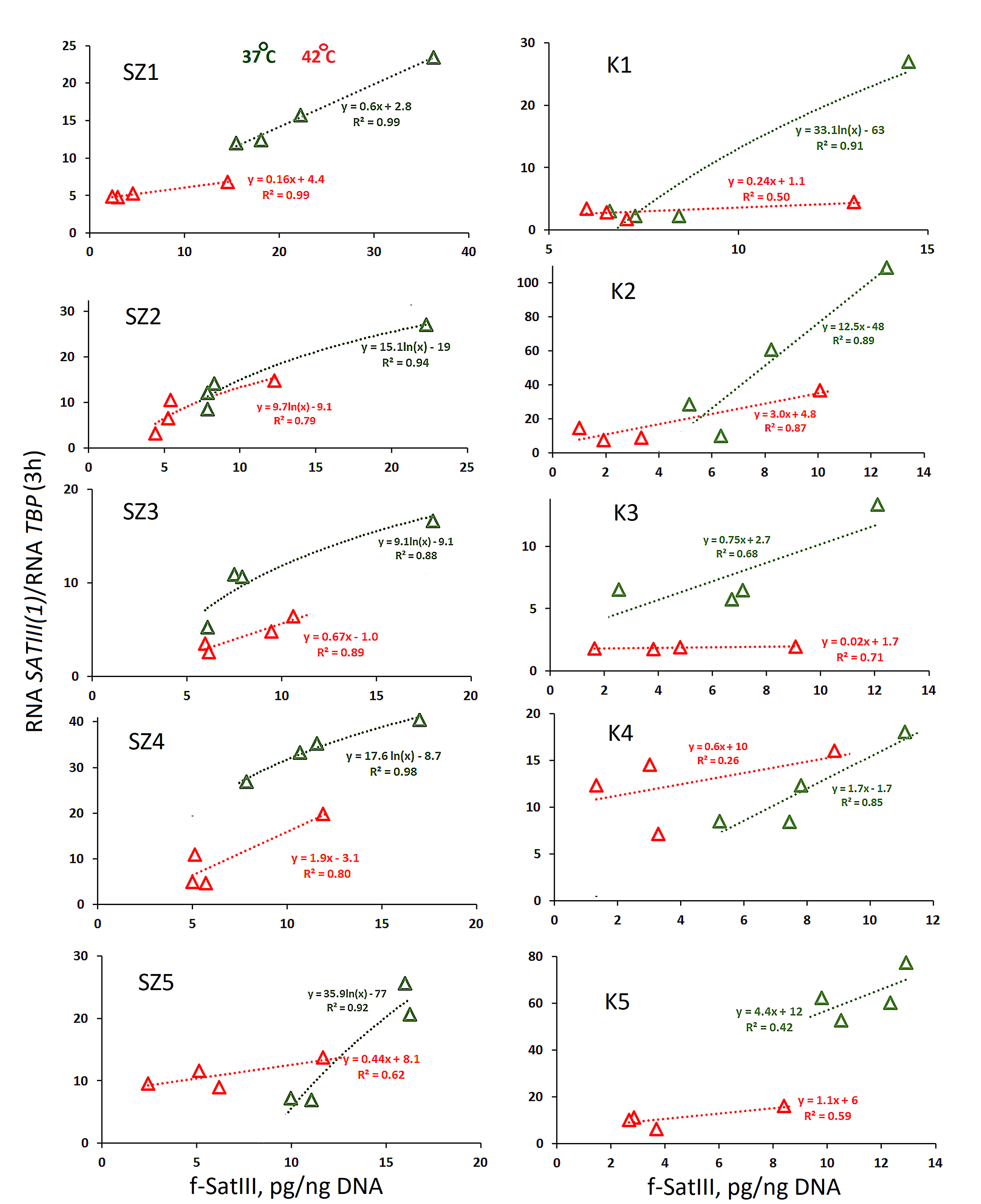

Supplement: Supplementary file 1 [file ijms-24-11283-s001.zip › Figure-4 Suppl.tif]
